# Does Curved Walking Sharpen the Assessment of Gait Disorders? An Instrumented Approach Based on Wearable Inertial Sensors

**DOI:** 10.3390/s20185244

**Published:** 2020-09-14

**Authors:** Valeria Belluscio, Elena Bergamini, Marco Tramontano, Rita Formisano, Maria Gabriella Buzzi, Giuseppe Vannozzi

**Affiliations:** 1 Department of Movement, Human and Health Sciences, Interuniversity Centre of Bioengineering of the Human Neuromusculoskeletal System, University of Rome “Foro Italico”, Piazza Lauro de Bosis 15, 00135 Roma, Italy; valeria.belluscio@gmail.com (V.B.); m.tramontano@studenti.uniroma4.it or giuseppe.vannozzi@uniroma4.it (G.V.); 2 IRCCS Santa Lucia Foundation, Via Ardeatina 306, 00179 Roma, Italy; r.formisano@hsantalucia.it (R.F.); mg.buzzi@hsantalucia.it (M.G.B.)

**Keywords:** straight walking, curved trajectories, gait quality, body accelerations, figure of 8 walk test, traumatic brain injury, steering of locomotion, dynamic balance, mobility evaluation, turning

## Abstract

Gait and balance assessment in the clinical context mainly focuses on straight walking. Despite that curved trajectories and turning are commonly faced in our everyday life and represent a challenge for people with gait disorders. The adoption of curvilinear trajectories in the rehabilitation practice could have important implications for the definition of protocols tailored on individual’s needs. The aim of this study was to contribute toward the quantitative characterization of straight versus curved walking using an ecological approach and focusing on healthy and neurological populations. Twenty healthy adults (control group (CG)) and 20 patients with Traumatic Brain Injury (TBI) (9 severe, sTBI-S, and 11 very severe, sTBI-VS) performed a 10 m and a Figure-of-8 Walk Test while wearing four inertial sensors that were located on both tibiae, sternum and pelvis. Spatiotemporal and gait quality indices that were related to locomotion stability, symmetry, and smoothness were obtained. The results show that spatiotemporal, stability, and symmetry-related gait patterns are challenged by curved walking both in healthy subjects and sTBI-S, whereas no difference was displayed for sTBI-VS. The use of straight walking alone to assess gait disorders is thus discouraged, particularly in patients with good walking abilities, in favor of the adoption of complementary tests that were also based on curved paths.

## 1. Introduction

Curved trajectories and changes in path directions (i.e., turning a corner or avoiding obstacles) are commonly faced in our everyday life [1]. During daily activities, in fact, 20–50% of all steps are turns [1] and the ability to successfully perform these turning/changes is considered to be fundamental for a safe locomotion [2]. When compared to straight walking, largely considered an automatic task for the symmetry of body movements which facilitates segment coordination [3], turning has been identified as more challenging, since it involves cognitive to motor adaptations, which are necessary to perform a smooth trajectory toward the new direction [2,4]. Among these adaptations, the following are noteworthy: the forward leg assumes the role of the axis of rotation; temporal and spatial features of the movement of the inner and outer leg, as well as foot rotations, become asymmetric; the body center of mass shifts to the inner leg creating a torque on the frontal plane that counteracts the centrifugal acceleration, while the head anticipates body yaw of almost 200 milliseconds [3,4]. In addition, it has been observed that healthy subjects adapt their locomotor velocity to the radius of curvature of the path they are following, with the central nervous system adapting the motor strategy by taking the geometry of the path into account [5].

If these adaptations can be considered trivial for healthy people, they represent an actual challenge for the elderly [1,6,7] and for patients with neurological disorders [8,9,10,11]. Furthermore, even if it is not clear if falls are more frequent during turns, it has been shown that falling during a turn has an increased risk of serious injury [12], and that staggering when turning is a prominent characteristic of recurrent fallers [13].

Despite these evidences, very often, the assessment of gait and balance in the clinical context does not adequately reflect mobility function during daily life: motor function evaluation, in fact, usually focuses on straight walking tests, like the 10-m, 2-min, or 6-min walk test, or the Timed Up and Go test, where a single 180 degrees turn is performed [14].

Hess and colleagues [15] recently developed a test that is able to assess the ability to turn in clockwise and counterclockwise directions, involving both straight and curved paths, thus representing a more ecological assessment condition: the Figure-of-8 Walk Test (F8WT). Its reliability in assessing walking skills has been proved in people with stroke, showing the ability of this test in differentiating between healthy and pathological conditions [16]. The F8WT has been used to assess the efficacy of intervention programs for the elderly [17], with those who performed more complex rehabilitation programs exhibiting fewer steps and longer strides during the test. Curvilinear tests have been also used in people with Parkinson’s disease [8,9] and hemiparetic patients [10], highlighting gait abnormalities that were specifically related to the curved walking performance. However, the abovementioned literature mainly considered spatiotemporal parameters of gait (e.g., step count, stride length, mean velocity), without investigating the behavior of the upper-body, which is gaining interest among researchers and clinicians due to its fundamental role in the maintenance of up-right gait stability, symmetry, and smoothness of movement [18,19]. Furthermore, most of the studies focused on lab-based assessment, which lacks ecological validity and often entails cumbersome set up or limited acquisition volumes. In this respect, the possibility to perform patient’s assessment in a more familiar environment and to identify any functional impairment that may affect safety represents a crucial added value to the clinical routine and may be critical to the prevention of re-injury as well as of the planning of adequate rehabilitation strategies.

This is particularly true in those populations who commonly experience neuro-motor deficits. An example comes from people who suffered from a severe traumatic brain injury (sTBI), whose walking skills have been recently investigated using a wearable sensors-based approach in [18], highlighting that biomechanical parameters that are related to walking stability, symmetry, and smoothness are able to discriminate between different levels of severity within sTBI. Additionally, free-living mobility, thus both straight walking and turning, was investigated in people with mild TBI with wearable sensors [20]. The results of that study reported that free-living turn angle, velocity, duration, and variability were altered in patients with chronic TBI.

Because the use of a challenging walking modality, such as walking along a curved path, could have crucial implications for the definition of rehabilitation protocols tailored on individual’s needs, the purpose of this study was to provide a contribution in this direction. To this aim, straight and curved walking were quantitatively assessed in both healthy and sTBI populations using an instrumented approach that was based on inertial sensors. A set of biomechanical gait quality indices concerning stability, symmetry, and smoothness of movement were extracted and their behavior compared between the two different walking modalities, in both populations.

The hypothesis is that curved walking will put at strain motor control mechanisms more than straight walking. This is expected to be more evident in people who suffered from a sTBI and to depend on patient’s walking ability level, thus to the severity of the pathology.

## 2. Materials and Methods

This research study was performed at the IRCCS Santa Lucia Foundation in Rome and has been approved by the Local Independent Ethics Committee of IRCCS Santa Lucia Foundation (protocol number: CE/PROG.700). Every participant read and signed an informed consent form.

### 2.1. Participants

Twenty people who suffered from a sTBI, 12 males and 8 females, were involved in the study. To be included in the study, participants needed to have: (*i*) age between 15–65 years; (*ii*) a Glasgow Coma Scale (GCS) score ≤ 8 (used to objectively describe the severity of impaired consciousness at the time of injury) [21]; (*iii*) a Level of Cognitive Functioning (LCF) ≥ 7 [22]; (*iv*) presence of disturbances in static and dynamic balance. Furthermore, it was necessary that they were able to understand verbal commands.

From a large convenience sample of healthy subjects (control group, CG), 20 healthy subjects who matched sTBI patients for age and gender were selected, after careful collection of medical history in order to exclude those who reported the presence of any orthopedic, neurological, or other co-morbidities that could have influenced the motor performance. Non-corrected visual deficits were exclusion criteria for both groups. The proposed sample size complied with the minimum number of participants recommended by a power analysis purposely performed (α = 0.05; power (1 − β) = 0.92, effect size d: 0.5) for non-parametric comparisons [23].

According to their score in the Dynamic Gait Index (DGI) Clinical Scale, sTBI patients were further divided into two sub-groups: this further division allowed codifying different levels of walking ability and assessing a patient’s ability to modify gait in response to changing task demands [24]: persons with a score ≥19 were considered severe (9 people, sTBI-S), while those with a score < 19 were considered as very severe (11 people, sTBI-VS), according to [25]. Table 1 reports demographic characteristics of each sub-group.

### 2.2. Procedures

**Figure 1:**
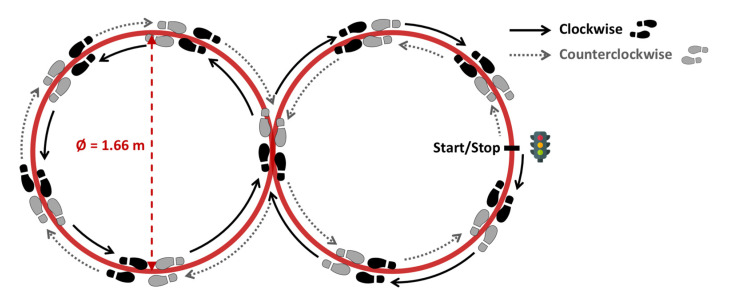
The Figure-of-8 Walk Test, adapted from [18].

All participants were asked to walk under two different walking conditions, straight and curved, respectively, performing a 10 m Walk Test (10 mWT) and a Figure-of-8 Walk Test (F8WT). During the 10 mWT, the participants were asked to walk on a straight 14-meter-long walkway for three times. Hence, in order to assess steady-state walking, only the middle 10 m (marked with tape on the floor) were considered for further analyses. For the F8WT, the participants were asked to walk a figure-of-8 shaped path (Figure 1) marked with tape on the floor [18], consisting of two circles of 1.66 m (5.44 ft) of diameter each. The task was performed three times both clockwise and counterclockwise. Linear and curved tasks were both randomly performed at participants’ preferred walking pace and, for sTBI, a physiotherapist walked close to each patient to prevent falls.

### 2.3. Equipment

Each participant was equipped with four wearable inertial measurement units (IMUs) (128 Hz, Opal, APDM, Portland, OR, USA), automatically synchronized through the Mobility Lab software. A 10 μs resolution among the IMU network is guaranteed from the vendor. All units had exactly the same technical characteristics and measured three-dimensional (3D) linear acceleration and angular velocity with respect to a case-aligned system of reference (±6 g with g = 9.81 m·s^2^, and ±1500 deg·s^−1^ of full-range scale, respectively). Two IMUs were located on the center of the sternum (S), and at L4/L5 level, slightly above the pelvis (P), and other two IMUs were positioned on both distal tibiae, slightly above the lateral malleoli. The upper-body units were used to assess the upper-body stability, whereas the tibiae-mounted ones for stride segmentation. The IMUs were positioned by two expert physiotherapists who securely fixed the devices following ad hoc instructions relying on specific anatomical landmarks. Each IMU was attached to the participants’ body with ad hoc Velcro straps.

### 2.4. Data Processing

The data were processed through ad hoc scripts and functions implemented using the Matlab^®^ software (The MathWorks Inc., Natick, MA, USA). First, the two IMUs located on the upper body were verticalized through a rigid transformation applied during the static phase before each trial. This transformation was obtained using the accelerometer as an inclinometer and thus obtaining both IMUs inclination with respect to gravity. [26]. The resulting IMU axes were considered to approximate the antero-posterior (AP), medio-lateral (ML), and cranio-caudal (CC) anatomical axes. This procedure guaranteed identical starting conditions for all the participants as well as a reliable and repeatable system of reference for both sternum and pelvis IMUs.

The following spatiotemporal parameters and gait quality indices were then obtained for both the 10 mWT and F8WT:

- Average walking speed (WS): total distance/time to complete the test.
- Average stride duration (SD): time to complete the test/total number of strides.
- Stride frequency (SF) total number of strides/time to complete the test.
- Stability:
  *(i)*  normalized Root Mean Square (*nRMS*) values of the measured accelerations at P and S levels, calculated by dividing the *RMS* obtained for the AP and ML components by the *RMS* of the CC component. As widely reported in the literature, the greater the *nRMS* values, the higher the amount of acceleration and, hence, the higher the instability [27];
    $$RMS_{j}K=\frac{1}{N}\sqrt{\sum_{i=1}^{N}a_{i}^{2}};\;nRMS_{j}K=\frac{RMS_{j}K}{RMS_{CC}K}$$
    where *j* is the component (AP and ML), K is the upper body level (P and S), *N* is the number of data sample, and a is the acceleration signal;
  *(ii)*  attenuation coefficient (AC) [28] between P and S levels, for the acceleration component (*j*), defined as:
    $$ACPS_{j}=\left(1-\frac{RMS_{j}S}{RMS_{j}P}\right)\cdot 100$$
    A positive coefficient represents an attenuation of the accelerations from lower to upper body levels, whereas a negative coefficient indicates an amplification.
- Symmetry: improved Harmonic Ratio (iHR) calculated for each acceleration component (*j*) measured at the pelvis level, as proposed by [29]. It was calculated as:
  $${\mathrm{iHR}}_{j}=\frac{\sum \mathrm{Power}\;\mathrm{of}\;\mathrm{intrinsic}\;\mathrm{harmonics}}{\sum \mathrm{Power}\;\mathrm{of}\;\mathrm{intrinsic}\;\mathrm{harmonics}+\sum \mathrm{Power}\;\mathrm{of}\;\mathrm{extrinsic}\;\mathrm{harmonics}}\cdot 100$$
  where an iHR of 0% is considered as total asymmetry and 100% as perfect symmetry. According to [29], the number of considered harmonics was 20 and the number of considered strides for each participant was greater than 20.
- Smoothness: SPectral ARC length (SPARC) [30] calculated for each acceleration component (*j*) measured at the P level. It estimates smoothness by calculating the arc length of the Fourier magnitude spectrum of a given signal profile within a defined frequency range. The calculation of SPARC has been performed, as follows:
  $$-\int_{0}^{{\tilde{\omega }}_{c}}[{\left(\frac{1}{{\tilde{\omega }}_{c}}\right)}^{2}+{\left(\frac{d\hat{A}\left(\tilde{\omega }\right)}{d\tilde{\omega }}{)}^{2}\right]}^{\frac{1}{2}}d\tilde{\omega };\hat{A}\left(\tilde{\omega }\right)=\frac{A\left(\tilde{\omega }\right)}{A\left(0\right)}$$
  $${\tilde{\omega }}_{c}=\min\{{{\tilde{\omega }}_{c}}^{\max},\min\{\tilde{\omega },\hat{A}\left(r\right)<\bar{A}\;\forall r>\tilde{\omega }\}\}$$
  where A ($\tilde{\omega }$) is the Fourier magnitude spectrum of the acceleration signal and $\hat{A}$($\tilde{\omega }$) is the normalized magnitude spectrum. ${\tilde{\omega }}_{c}$ is adaptively selected based on a given threshold $\bar{A}$ and is upper bound by ${{\tilde{\omega }}_{c}}^{\max}$. The higher the SPARC value, the smoother the movement.

### 2.5. Statistical Analysis

The IBM SPSS Statistics software (v23, IBM Corp., Armonk, NY, USA) was used to perform both descriptive and inferential statistical analyses. The normal distribution of each parameter was verified using the Shapiro–Wilk test. As most of the parameters were not normally distributed, the Wilcoxon test was performed to evaluate differences between linear and curved walking-based parameters in each sub-group (sTBI-VS, sTBI-S, CG). The alpha level of significance was set at 0.05.

## 3. Results

### 3.1. Spatiotemporal Parameters

**Figure 2:**
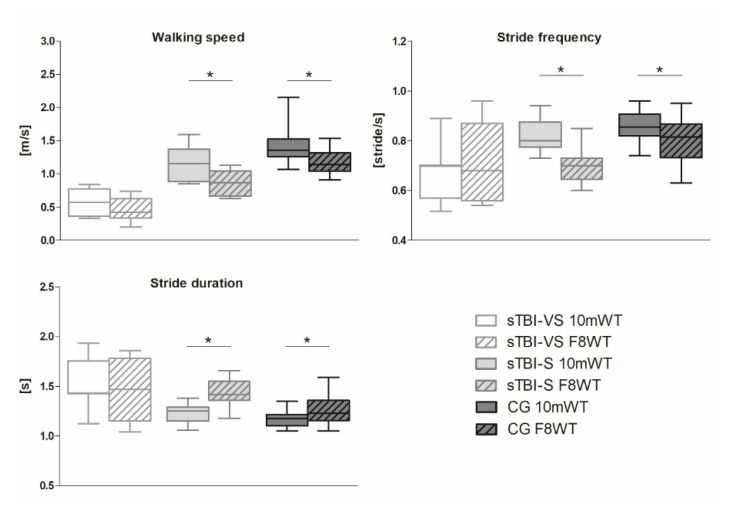
Whisker’s plots reporting walking speed, stride frequency, and stride duration for both the 10 mWT and the F8WT in sTBI-VS, sTBI-S, and CG. In all subplots, the horizontal lines with asterisks indicate statistically significant differences.

Figure 2 reports the spatiotemporal parameters results. In details, sTBI-S and the CG show differences between straight and curved walking in terms of walking speed (*p* = 0.027 for sTBI-S and *p* = 0.018 for CG), stride frequency (*p* = 0.021 for sTBI-S and *p* = 0.010 for CG), and stride duration (*p* = 0.021 for sTBI-S and *p* = 0.012 for CG), and, whereas sTBI-VS presents no differences in the spatiotemporal parameters when comparing the two different walking modalities.

### 3.2. Gait Quality Indices

**Figure 3:**
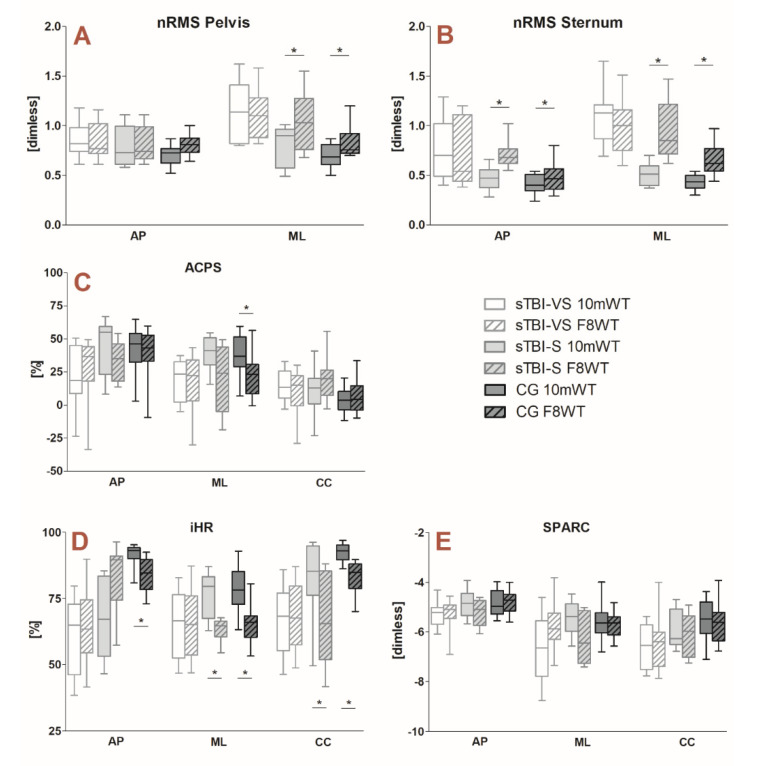
Whisker’s plots reporting gait quality indices for both sTBI sub-groups (sTBI-VS and sTBI-S) and for CG in the 10 mWT (solid boxes) and F8WT (striped boxes). Panel (**A**,**B**): normalized RMS values (nRMS) for pelvis and sternum levels, respectively; Panel (**C**): attenuation coefficients (AC); Panel (**D**): improved Harmonic Ratio (iHR); Panel (**E**): SPectral ARC length (SPARC). AP, antero-posterior; ML, medio-lateral; CC, cranio-caudal; P, pelvis; S. In all plots, the horizontal lines with asterisks indicate statistically significant differences.

When considering gait stability, symmetry, and smoothness of movement, sTBI-S and CG showed significant differences between linear and curved walking, whereas no difference was displayed for sTBI-VS. Figure 3 reports detailed results.

## 4. Discussion

The aim of the present study was to provide a contribution toward the quantitative characterization of straight versus curved walking using an ecological approach and focusing on both healthy and neurological populations. A wearable sensor-based protocol was adopted directly in-the-field, allowing to obtain information regarding participants’ gait quality. Specifically, gait stability, symmetry, and smoothness-related indices were obtained during a 10 m Walk Test (10 mWT) and a Figure-of-8 Walk Test (F8WT), in both healthy people (CG) and patients with severe and very severe Traumatic Brain Injury (sTBI-S and sTBI-VS, respectively).

The results show that significant differences between straight and curved paths exist for both spatiotemporal and gait quality indices in CG and sTBI-S, whereas no statistical difference exists between the two walking modalities in sTBI-VS, indicating that this group of patients presents similar biomechanical patterns in the two conditions. This is in agreement with the existing literature focusing on straight versus curved walking and confirms that: (i) curved walking requires different adaptations (with respect to straight walking) in order to fit the biomechanical constraints imposed by linear and rotational dynamics of turning [2,31]; (ii) since these adaptations rely on the central nervous system which, during curved walking, takes over the control of locomotion at the expenses of the spinal automatisms [31], people with neurological disorders might encounter difficulties in managing the interplay between balance control and center of mass progression when steering and turning [8]; and, (iii) the kind and entity of the abovementioned adaptations depend on walking velocity [2,31]: when walking speed is very low (below 0.9 m·s^−1^ during straight walking), differences in the spatiotemporal and gait quality-related parameters between straight and curved locomotion may become negligible [31].

Consistently, the very severe TBI sub-group considered in the present study was reported to walk at a very low speed (0.56 and 0.42 m·s^−1^ for the linear and curvilinear walking, respectively), likely as an expression of the motor deficit and to counteract the fear of falling, or because of post-traumatic parkinsonism features [32,33]. On the other hand, less severely affected patients displayed a median speed value of 1.15 m·s^−1^ during straight walking, which is higher than the abovementioned threshold [31]. Similar to healthy adults, in fact, they exhibited different walking patterns when performing straight and curvilinear paths. Specifically, consistent with what has been reported in previous literature [31], the more challenging gait modality induces a decrease in walking speed, an increase in stride duration, and a decrease in the stride frequency, with respect to the straight path (Figure 2). This confirms that human subjects adapt their locomotor velocity to the radius of curvature of the path they are following, with the velocity that tends to increase when the trajectory becomes straighter and decrease when it becomes more curved [5].

These conclusions are also strongly supported by the analysis of gait quality indices (related to stability, symmetry, and smoothness of movement), for which sTBI-VS subgroup did not show any difference between the two walking modalities (Figure 3). As reported above, this might be explained by the very low walking speed and patient’s walking ability level, which might determine a lack of neuromotor compensations strategies when going from a linear to a curvilinear path. A hypothesis is that very severe patients substantially perform straight and curved trajectories with a very similar approach, covering a curvilinear path as it was made by consecutive small straight paths. This seems also confirmed by the results obtained for gait symmetry parameters (Figure 3D): no significant difference was found between 10 mWT and F8WT in very severe patients, indicating that this group of patients does not present the intrinsic asymmetric motion of the lower limbs that was generally observed during curved walking [2,3,31].

Conversely, when considering gait quality indices in sTBI-S and CG, straight and curved walking-related parameters displayed overall differences, particularly in the medio-lateral (ML) direction (Figure 3). Specifically, concerning gait stability, both groups showed higher nRMS values in the ML direction at pelvis and sternum levels when performing the F8WT compared to the 10 mWT (Figure 3A,B). This is likely due to the arise of the centripetal acceleration, which is needed to counteract the centrifugal acceleration due to the curved trajectory [2,34]. At the sternum level (Figure 3B), these differences are even more evident and involve the antero-posterior (AP) component of the acceleration as well. This might be explained by the greater distance between the sternum and the contact foot, with respect to the pelvis, which determines increased linear velocity and acceleration, consistently with the hypothesis of a body modeled as an inverse pendulum during the stance phase. In healthy subjects, the increase in sternum accelerations during curved walking determines a significant decrease of the attenuation coefficient (AC) from the pelvis to the sternum (Figure 3C). Because higher nRMS and decreased AC values have been widely associated with decreased stability [18,27], the results of the present study confirm that dynamic balance stability could be a critical aspect in curved trajectories, more than it is during straight walking. Indeed, the shifting of the body center of mass toward the inner part of the curve (generating a medio-lateral torque necessary to counteract the centrifugal acceleration and, thus, to avoid going off the tangent [34]), often flanked to a trunk tilt in the same direction, may contribute to jeopardizing the control of balance, particularly in patients with neurological disorders [8,10]. These aspects are worth to be taken into account when designing rehabilitation protocols aimed at improving dynamic balance, restoring walking ability, and maintaining patients’ autonomy in everyday life.

As expected, statistically significant differences between the two walking modalities have also been found when considering gait symmetry (iHR) in both sTBI-S and CG (Figure 3D). As widely reported, intrinsic asymmetry characterizes steering and turning movements, being the spatial and temporal features of the inner and outer leg movement different [3,4]. It is interesting to note that, from straight to curved walking, the medio-lateral component of the iHR decreases from about 80% to 65% in both sTBI-S and CG (Figure 3D). In this respect, it must be considered that any change in the adjustment of the asymmetric step length of the inner and outer legs during curved walking produces important effects on the mechanics of locomotion [34]. Thus, specific and effective brain control is required for correct rotation of the lower limbs and the inversion or eversion of the ankle for successful placement of the foot on the ground [3,31]. The latter is a crucial aspect not only during heel strike, but also during the late stance phase. In fact, during heel strike, the medio-lateral distance between the center of mass and the center of pressure is maximal as well as the medio-lateral torque pushing the body toward the inner part of the curve, whereas during the late stance phase, the inner “fall” of the body due to the centripetal force is braked by the reduction of the torque lever arm caused by the shift of the center of pressure to the medial part of the outer foot [34]. These mechanisms, which characterize curved walking in healthy adults, may fail in people with neurological disorders and, again, should be considered when designing an effective rehabilitation protocol.

On the other hand, no differences have been found in terms of smoothness of movement (SPARC) (Figure 3E): smoothness of gait is a quality that reflects the continuousness or non-intermittency of walking [35]. In this case, the lack of differences between the two walking modalities in sTBI-VS might be explained observing that they performed an intermitted, segmented trajectory during both tasks. Conversely, smoothness in high functioning patients and healthy controls seems not to be influenced by the curvilinear trajectory.

The results of the present study suggest that in very severe TBI patients, who have more impaired walking ability, straight walking-based tests may provide useful information about the patient’s impairment and its evolution, with no need of moving to more complex walking modalities. On the other hand, tests based only on straight paths may not be always adequate to reveal motor-related disorders in patients with good walking abilities, such as sTBI-S. Curved walking-based tests, like the F8WT, indeed have the potential to reveal changes due to diseases of the central or peripheral nervous system [10,18], to the evolution of walking disorders or to the administration of rehabilitation protocols. In addition, attention should be devoted to the rehabilitation of curved walking in high-functioning patients with neuro-motor deficits in order to improve their autonomy and quality of life.

The presented results must be interpreted in light of the following considerations: in spite of the compliant number of participants, due to the heterogeneity of the sTBI sample mainly attributable to the severity/location of the trauma, a larger sample size would be necessary to further confirm the findings of the present work. The larger sample size would also allow for defining a threshold value useful to identify signs of disability in apparently healthy people or, for pathological populations, to derive the smallest difference in parameter scores that patients perceive as beneficial, namely the minimal clinically important difference (MCID). The determination of these threshold/MCID would be valuable not only for early detection of motor-related disorders, but also for the assessment and design of more effective therapies. Concerning sTBI patients’ variability, it is worth underlining that the results about parameters’ variability are in agreement with the existing literature [31], showing an increase in the variability (in terms of IQR) of spatiotemporal and most gait quality indices from straight to curved walking. No information was obtained about trunk inclination while walking along curvilinear paths or about upper limb movements, while they could provide details about lower-upper limbs coordination and upper body rigidity/stability. Therefore, further studies should take into consideration the evaluation of these aspects in order to provide a more complete overview of the investigated phenomena. 

## 5. Conclusions

The present findings show that spatiotemporal, stability, and symmetry-related gait patterns are challenged by curved walking both in healthy subjects and in patients who suffered from a sTBI, with different behavior of the latter according to trauma severity and walking ability level. This study promotes the adoption of in-field wearable protocols to support the assessment of locomotion in real life contexts in conjunction with home rehabilitation practices. The present results discourage the use of straight walking alone to assess gait disorders, in favor of the adoption of complementary tests based on both straight and curved paths, such as the Figure-of-8 Walk Test. Being more representative of the complex motor skills that are required to adapt to changes in real-life situations, the use of curved walking in both assessment and rehabilitation practice has the potential to reveal crucial information about diseases evolution and to improve patients’ balance, safety, autonomy, and quality of life. In conclusion, the present study provides quantitative information about straight and curved walking strategies in healthy and neurological populations, supporting the clinical staff in proposing sequential and propaedeutic rehabilitative approaches tailored on patient’s walking abilities.

## Figures and Tables

**Table 1:** Demographic and anthropometric characteristics of very severe Traumatic Brain Injury (sTBI-VS), severe Traumatic Brain Injury (sTBI-S), and control group (CG). Median and interquartile (IQR) values are displayed. Groups were homogeneous in terms of age, body mass, and height (*p* > 0.05). sTBI-S and sTBI-VS did not show statistical differences in terms of time since trauma (*p* > 0.05).

	sTBI-VS	sTBI-S	CG
**Nr. of participants**	11	9	20
**Nr. of males**	7	5	12
**Age (ears)**	32.0 (9.7)	34.1 (9.1)	33.6 (10.8)
**Time since trauma (days)**	504 (456)	302 (178)	-
**Body mass (kg)**	72.0 (13.7)	75.9 (16.2)	78.3 (14.9)
**Body height (m)**	1.74 (0.02)	1.73 (0.04)	1.78 (0.05)

## References

[1] Segal A.D., Orendurff M.S., Czerniecki J.M., Shofer J.B., Klute G.K. (2008). Local dynamic stability in turning and straight-line gait. J. Biomech..

[2] Courtine G., Schieppati M. (2003). Human walking along a curved path I. Body trajectory, segment orientation and the effect of vision. Eur. J. Neurosci..

[3] Courtine G., Schieppati M. (2004). Tuning of a basic coordination pattern constructs straight-ahead and curved walking in humans. J. Neurophysiol..

[4] Hase K., Stein R.B. (1999). Turning strategies during human walking. J. Neurophysiol..

[5] Vieilledent S., Kerlirzin Y., Dalbera S., Berthoz A. (2001). Relationship between velocity and curvature of a human locomotor trajectory. Neurosci. Lett..

[6] Dite W., Temple V.A. (2002). Development of a clinical measure of turning for older adults. Am. J. Phys. Med. Rehabil..

[7] King L., Mancini M., Salarian A., Holmstrom L., McNames J., Horak F.B. (2013). Mobility lab to assess balance and gait with synchronized body-worn sensors. J. Bioeng. Biomed. Sci..

[8] Turcato A.M., Godi M., Giardini M., Arcolin I., Nardone A., Giordano A., Schieppati M. (2018). Abnormal gait pattern emerges during curved trajectories in high-functioning Parkinsonian patients walking in line at normal speed. PLoS ONE.

[9] Guglielmetti S., Nardone A., de Nunzio A.M., Godi M., Schieppati M. (2009). Walking along circular trajectories in Parkinson’s disease. Mov. Disord..

[10] Godi M., Nardone A., Schieppati M. (2010). Curved walking in hemiparetic patients. J. Rehabil. Med..

[11] Fino P.C., Nussbaum M.A., Brolinson P.G. (2016). Locomotor deficits in recently concussed athletes and matched controls during single and dual-task turning gait: Preliminary results. J. Neuroeng. Rehabil..

[12] El-Gohary M., Pearson S., McNames J., Mancini M., Horak F., Mellone S., Chiari L. (2014). Continuous monitoring of turning in patients with movement disability. Sensors.

[13] Tinetti M.E. (1986). Performance-oriented assessment of mobility problems in elderly patients. J. Am. Geriatr. Soc..

[14] Higashi Y., Yamakoshi K., Fujimoto T., Sekine M., Tamura T. (2008). Quantitative evaluation of movement using the timed up-and-go test. IEEE Eng. Med. Biol. Mag..

[15] Hess R.J., Brach J.S., Piva S.R., vanSwearingen J.M. (2010). Walking skill can be assessed in older adults: Validity of the Figure-of-8 Walk Test. Phys. Ther..

[16] Wong S.S.T., Yam M.S., Ng S.S.M. (2013). The Figure-of-Eight Walk test: Reliability and associations with stroke-specific impairments. Disabil. Rehabil..

[17] Song H.S., Kim J.Y. (2015). The effects of complex exercise on walking ability during direction change and falls efficacy in the elderly. J. Phys. Ther. Sci..

[18] Belluscio V., Bergamini E., Tramontano M., Bustos A.O., Allevi G., Formisano R., Vannozzi G., Buzzi M.G. (2019). Gait quality assessment in survivors from severe traumatic brain injury: An instrumented approach based on inertial sensors. Sensors.

[19] Bergamini E., Iosa M., Belluscio V., Morone G., Tramontano M., Vannozzi G. (2017). Multi-sensor assessment of dynamic balance during gait in patients with subacute stroke. J. Biomech..

[20] Stuart S., Parrington L., Martini D.N., Kreter N., Chesnutt J.C., Fino P.C., King L.A. (2020). Analysis of free-living mobility in people with mild traumatic brain injury and healthy controls: Quality over quantity. J. Neurotrauma.

[21] Teasdale G.M. (1995). Head injury. J. Neurol. Neurosurg. Psychiatry.

[22] Ciurli P., Bivona U., Barba C., Onder G., Silvestro D., Azicnuda E., Rigon J., Formisano R. (2010). Metacognitive unawareness correlates with executive function impairment after severe traumatic brain injury. J. Int. Neuropsychol. Soc..

[23] Cohen J. (1992). Statistical power analysis. Curr. Dir. Psychol. Sci..

[24] Herman T., Inbar-Borovsky N., Brozgol M., Giladi N., Hausdorff J.M. (2010). Dynamic Gait index in healthy older adults: The role of stair climbing, fear of falling and gender. Gait Posture.

[25] Shumway-Cook A., Baldwin M., Polissar N.L., Gruber W. (1997). Predicting the probability for falls in community-dwelling older adults. Phys. Ther..

[26] Bergamini E., Ligorio G., Summa A., Vannozzi G., Cappozzo A., Sabatini A.M. (2014). Estimating orientation using magnetic and inertial sensors and different sensor fusion approaches: Accuracy assessment in manual and locomotion tasks. Sensors.

[27] Kavanagh J.J., Menz H.B. (2008). Accelerometry: A technique for quantifying movement patterns during walking. Gait Posture.

[28] Mazzà C., Iosa M., Pecoraro F., Cappozzo A. (2008). Control of the upper body accelerations in young and elderly women during level walking. J. Neuroeng. Rehabil..

[29] Pasciuto I., Bergamini E., Iosa M., Vannozzi G., Cappozzo A. (2017). Overcoming the limitations of the Harmonic Ratio for the reliable assessment of gait symmetry. J. Biomech..

[30] Balasubramanian S., Melendez-Calderon A., Roby-Brami A., Burdet E. (2015). On the analysis of movement smoothness. J. Neuroeng. Rehabil..

[31] Godi M., Giardini M., Schieppati M. (2019). Walking along curved trajectories. Changes with age and Parkinson’s disease. Hints to rehabilitation. Front. Neurol..

[32] Tramontano M., Bergamini E., Iosa M., Belluscio V., Vannozzi G., Morone G. (2018). Vestibular rehabilitation training in patients with subacute stroke: A preliminary randomized controlled trial. NeuroRehabilitation.

[33] Formisano R., Zasler N.D. (2014). Posttraumatic parkinsonism. J. Head Trauma Rehabil..

[34] Turcato A.M., Godi M., Giordano A., Schieppati M., Nardone A. (2015). The generation of centripetal force when walking in a circle: Insight from the distribution of ground reaction forces recorded by plantar insoles. J. Neuroeng. Rehabil..

[35] Balasubramanian S., Melendez-Calderon A., Burdet E. (2012). A robust and sensitive metric for quantifying movement smoothness. IEEE Trans. Biomed. Eng..

